# CD5 blockade, a novel immune checkpoint inhibitor, enhances T cell anti-tumour immunity and delays tumour growth in mice harbouring poorly immunogenic 4T1 breast tumour homografts

**DOI:** 10.3389/fimmu.2024.1256766

**Published:** 2024-02-29

**Authors:** Faizah M. Alotaibi, Wei-Ping Min, James Koropatnick

**Affiliations:** ^1^ College of Science and Health Professions, King Saud Bin Abdulaziz University for Health Sciences, Alahsa, Saudi Arabia; ^2^ King Abdullah International Medical Research Center, Ministry of National Guard-Health Affairs, Riyadh, Saudi Arabia; ^3^ Department of Oncology, The University of Western Ontario, London, ON, Canada; ^4^ Department of Microbiology and Immunology, The University of Western Ontario, London, ON, Canada; ^5^ Cancer Research Laboratory Program, London Regional Cancer Program, Lawson Health Research Institute, London, ON, Canada

**Keywords:** CD5, immune checkpoint inhibitors, immunotherapy, cancer, T cell, drug

## Abstract

CD5 is a member of the scavenger receptor cysteine-rich superfamily that is expressed on T cells and a subset of B cells (B1a) cell and can regulate the T cell receptor signaling pathway. Blocking CD5 function may have therapeutic potential in treatment of cancer by enhancing cytotoxic T lymphocyte recognition and ablation of tumour cells. The effect of administering an anti-CD5 antibody to block or reduce CD5 function as an immune checkpoint blockade to enhance T cell anti-tumour activation and function *in vivo* has not been explored. Here we challenged mice with poorly immunogenic 4T1 breast tumour cells and tested whether treatment with anti-CD5 monoclonal antibodies (MAb) *in vivo* could enhance non-malignant T cell anti-tumour immunity and reduce tumour growth. Treatment with anti-CD5 MAb resulted in an increased fraction of CD8^+^ T cells compared to CD4^+^ T cell in draining lymph nodes and the tumour microenvironment. In addition, it increased activation and effector function of T cells isolated from spleens, draining lymph nodes, and 4T1 tumours. Furthermore, tumour growth was delayed in mice treated with anti-CD5 MAb. These data suggest that use of anti-CD5 MAb as an immune checkpoint blockade can both enhance activation of T cells in response to poorly immunogenic antigens and reduce tumour growth *in vivo*. Exploration of anti-CD5 therapies in treatment of cancer, alone and in combination with other immune therapeutic drugs, is warranted.

## 1 Introduction

CD5 is a type 1 transmembrane glycoprotein and a member of the scavenger receptor cysteine-rich superfamily expressed on T cells and a subset of B cells (B1a) (1). It can be detected early in the “double-negative” stage of T cell development and its level increases during T cell development (2). CD5 co-localises with TCR during the immunological synapse with antigen-presenting cells and regulates TCR signaling and promotes development of high-affinity antigen binding (3). In non-solid tumours, the majority of T and B cell malignancies are CD5-positive (4). Therefore, it has been used as a targetable tumour antigen for T and B cell malignancies (5). Several passive and active immunotherapeutic approaches have implemented the use of anti-CD5 immunoconjugates linked to cytotoxic molecules (6–12) and CD5 CAR T cells (13–22) to treat CD5^+^ hematologic malignancies.

On the other hand, strategies to target CD5 on immune cells rather than tumour cells themselves is not well-investigated. Nevertheless, current evidence suggests that this may be a useful therapeutic approach. When solid B16F10 syngeneic tumour homografts were grown in CD5 knockout mice, those mice exhibited increased anti-tumour immunity and delayed tumour growth compared to tumours grown in wild type mice (23). Furthermore, we have reported that differential CD5 levels among T cells in tumours and lymphoid organs can be associated with different levels of T cell activation and effector function (24). In addition, mice with transgenic expression of soluble human CD5 had delayed B16F10 tumour homograft growth compared to control mice (25). Because CD5 is also a ligand for CD5 (26), the sCD5 may act to block CD5 from binding to the TCR/CD3 complex and reduce the ability of CD5 to impair TCR signaling capable of activating T cells. Furthermore, tumour-infiltrating lymphocytes with low CD5 expression exhibited high anti-tumor activity compared to cells with CD5 high expression (24, 27). These results suggest that reducing CD5 function could result in increase anti-tumour activity and enhance immune activation.

In this study we investigated the capacity of anti-CD5 MAb to enhance T cell anti-tumour immunity. We administrated blocking, non-depleting anti-CD5 MAb in mice challenged with poorly immunogenic CD5-negative 4T1 mouse breast tumour cell homografts and investigated the effect on immune T cell activation and function and tumour growth. The data show that *in vivo* anti-CD5 MAb treatment enhanced T cell anti-tumour immunity and delayed tumour growth. These results suggest the therapeutic potential of using anti-CD5 MAb as an immune checkpoint blockade to promote anti-tumour T cell immunity.

## 2 Materials and methods

### 2.1 Mice and cells

Female BALB/c mice were purchased from The Jackson Laboratories (Jackson Laboratories, Bar Harbor, ME). All animals were between 8 and 12 weeks of age and housed in the Animal Care and Veterinary Services Facility at the Victoria Research Building, Lawson Health Research Institute, according to guidelines of the Canadian Council for Animal Care and under the supervision of the Animal Use Subcommittee of the University of Western Ontario. 4T1 mouse breast mouse tumour cells were purchased from the American Type Culture Collection (ATCC, Manassas, VA), and cultured in Dulbecco modified Eagle medium supplemented with 10% fetal bovine serum (FBS)(Invitrogen). All cells were kept at 37°C in 5% CO2. 4T1 tumour cells were counted by Coulter counter and resuspended into sterile PBS for further experiments.

### 2.2 *In vivo* treatment design

This experiment is designed to assess the impact of anti-CD5 MAb and tumour growth. To assess tumour growth after treatment, mice were injected subcutaneously with 5000 4T1 tumour cells on day 0. Mice were then randomly divided into two groups and received one of the following treatments by peritumoural injection: Group 1: isotype control (Anti-fluorescein mouse IgG2A, Fc, Silent™, Kappa, [Ab00102-2.3]; Absolute Antibody, Ltd, Oxford, UK), 25 μg/mouse on day 0 and every three to four days thereafter for a total of 11 injections. Group 2: anti-CD5 Mab (Anti-CD5 IgG2a, Fc, Silent™, Kappa, [Ab00208-2.3]; Absolute Antibody Ltd., Oxford UK), 25 μg/mouse on day 0 and every three to four days thereafter for a total of 11 injections.

### 2.3 Animal health

To determine the safety and efficacy of anti-CD5 Mab *in vivo*, mice were injected with anti-CD5 Mab (200 μg/mouse) on day 7 post subcutaneously tumour injection (50000 cells) and every three to four days thereafter for a total of four injections. Mice were monitored daily for potential adverse effects of tumour growth and/or antibody injection by qualified animal care technicians in the Animal Care and Veterinary Services Facility. When tumours reached the endpoint, mice were euthanized and tumour-infiltrating lymphocytes (TILs), spleens, and draining lymph nodes (DLN) were collected for immune profiling.

### 2.4 Preparation of splenocytes, lymphocytes and tumour infiltrating lymphocytes

Mice were euthanized when tumour reach 1500 mm^3^ and splenocytes, lymphocytes, and tumour-infiltrating lymphocytes (TILs) were obtained from tissues using a modification of our previously-reported method (28). Briefly, single cell suspensions of lymphocytes were obtained from mice by pressing spleens or lymph nodes through a 70 μm Falcon Cell Strainer (VWR, Mississauga, ON) into RPMI 1640 medium (GIBCO). Cells were then centrifuged (300xg, 10 mins, 4°C), and erythrocytes were lysed using Ammonium-Chloride-Potassium (ACK) red cell lysis buffer. The resulting live (trypan blue-negative) splenocytes and lymphocytes were counted manually by microscopy after dropping onto a glass slide. Cells obtained were stained for flow cytometric analysis as described below. TILs were obtained from freshly-resected tumour lesions, which were isolated immediately after mice euthanization. Tumours were cut into 2-3 mm^3^ fragments, and each tumour fragment was placed into an individual well of a 6 well plate and incubated in 2 ml of an enzyme digest mix consisting of RPMI1640 complete media containing 5% fetal bovine serum (FBS)(Invitrogen) and 10 mg ml^−1^ collagenase A (all from Sigma-Aldrich, Gillingham, UK) and incubated for 2 hours at room temperature under continuous rotation. Cells were then centrifuged (300xg, 10 mins, 4°C), and erythrocytes were lysed using Ammonium-Chloride-Potassium (ACK) red cell lysis buffer. The resulting live (trypan blue-negative) TILs were counted manually by microscopy after dropping onto a glass slide. Cells obtained were stained for flow cytometric analysis as described below.

### 2.5 Flow cytometry

To assess the levels of CD69, CD107a, CD137 and FasR on T cells by flow cytometry, lymphocytes obtained as described above were stained with the following antibodies: Brilliant Violet 711™ anti-mouse CD3 (BioLegend, San Diego, CA), Alexa Fluor^®^ 700 anti-mouse CD4 (BioLegend, San Diego, CA), PerCP/Cyanine5.5 anti-mouse CD8a (BioLegend, San Diego, CA), FITC Rat Anti-Mouse CD5 (BD Biosciences), PE Hamster Anti-Mouse CD69 (BD Biosciences), PE anti-mouse CD95 (Fas) Antibody (BioLegend, San Diego, CA), Brilliant Violet 421™ anti-mouse CD107a (LAMP-1) (BioLegend, San Diego, CA), PE anti-mouse CD137 (BioLegend, San Diego, CA). All flow cytometric analyses were performed as described previously (29) using appropriate isotype controls (Biolegend, San Diego, CA). Flow cytometry was performed using a BD™ LSR II Flow Cytometer (BD Biosciences) and data analyzed using Flowjo software (BD Bioscience). To assess the level of the indicated markers, organs were collected from tumour-bearing mice when mice were euthanized at the end of tumour growth. Cells were prepared as previously described (29, 30), and 2X10^5^ cells were stained and analyzed by flow cytometry as described above. Cells were treated with purified anti-mouse CD16/32 antibody (Clone 93) (Biolegend, San Diego, CA) for 15 min at 21°C in the dark to block CD16/CD32 interactions with the Fc domain of immunoglobulins. Cells were then stained with appropriate antibodies for 25 mins on ice in the dark, washed twice with FACS staining buffer, suspended in 0.5 ml FACS staining buffer, and analyzed by flow cytometry.

### 2.6 Statistical analysis

Statistical differences were assessed using a Student’s unpaired one-tailed t-test (GraphPad Prism 8.2.1). Data points indicate means of n values ± standard deviation (SD). Differences between data sets where p ≤ 0.05 were considered to be significant. Asterisks represent statistical significance.

## 3 Results

### 3.1 Animal health

No difference in mean animal weights between the isotype control MAb-treated group and the anti-CD5 MAb-treated group were observed (**Supplementary Figure 1**), and no overt adverse health effects (poor grooming, immobility, skin lesions, etc.) were observed in mice in either group.

### 3.2 Treatment with anti-CD5 MAb *in vivo* reduced 4T1 tumour growth in mice

**Figure 1:**
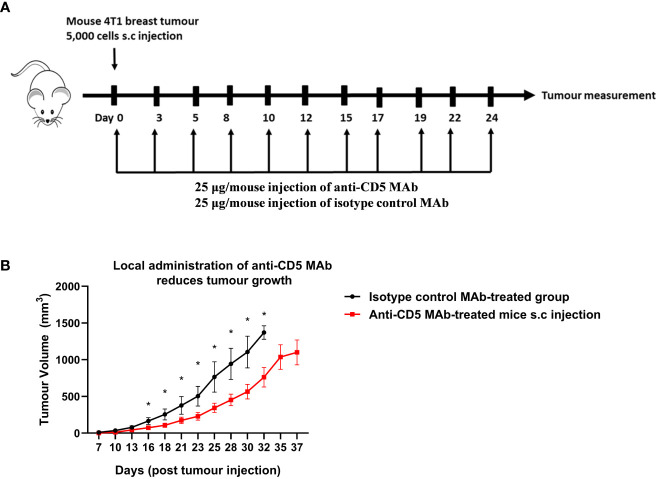
Treatment with anti-CD5 MAb delays 4T1 homograft tumour growth in host mice. 4T1 tumour-harbouring mice received 25 μg/mouse of anti-CD5 MAb on day 0 at the same time of subcutaneous injection of 4T1 tumour cells (every two days and over the course of 24 days) **(A)** Scheme for treatment plan. **(B)** Tumour volume. Data are mean ± SEM (n = 7 mice), one representative experiment of two, *p < 0.05 (Student’s unpaired one-tailed t-test).

The concentration of anti-CD5 MAb selected for repeated treatment (25 μg/mouse) was selected to avoid activation-induced T cell death (AICD). Preliminary experiments where mice were treated with 100 or 200 μg anti-CD5 MAb increased markers of T cell activation in spleens (increased CD69, fraction of CD8-positive T cells relative to CD4-positive T cells, etc.) but also increased activation-induced T cell death (AICD) as shown by increased Fas receptor in section 4 below. The lower concentration was therefore selected for treatment of tumour-bearin+g mice (**Figure 1A**). Mouse 4T1 breast tumour homograft growth was measured after treatment with anti-CD5 MAb. Tumours in mice treated with anti-CD5 MAb mice grew more slowly than in isotype control antibody-treated mice (**Figure 1B**). These data indicate that anti-CD5 MAb administration reduced 4T1 tumour growth in mice when administered *in vivo* and, as described in Section 1 (above), that the treatment had no overt adverse effects on mouse health.

### 3.3 Increased T cell activation after treatment with anti-CD5 MAb

**Figure 2:**
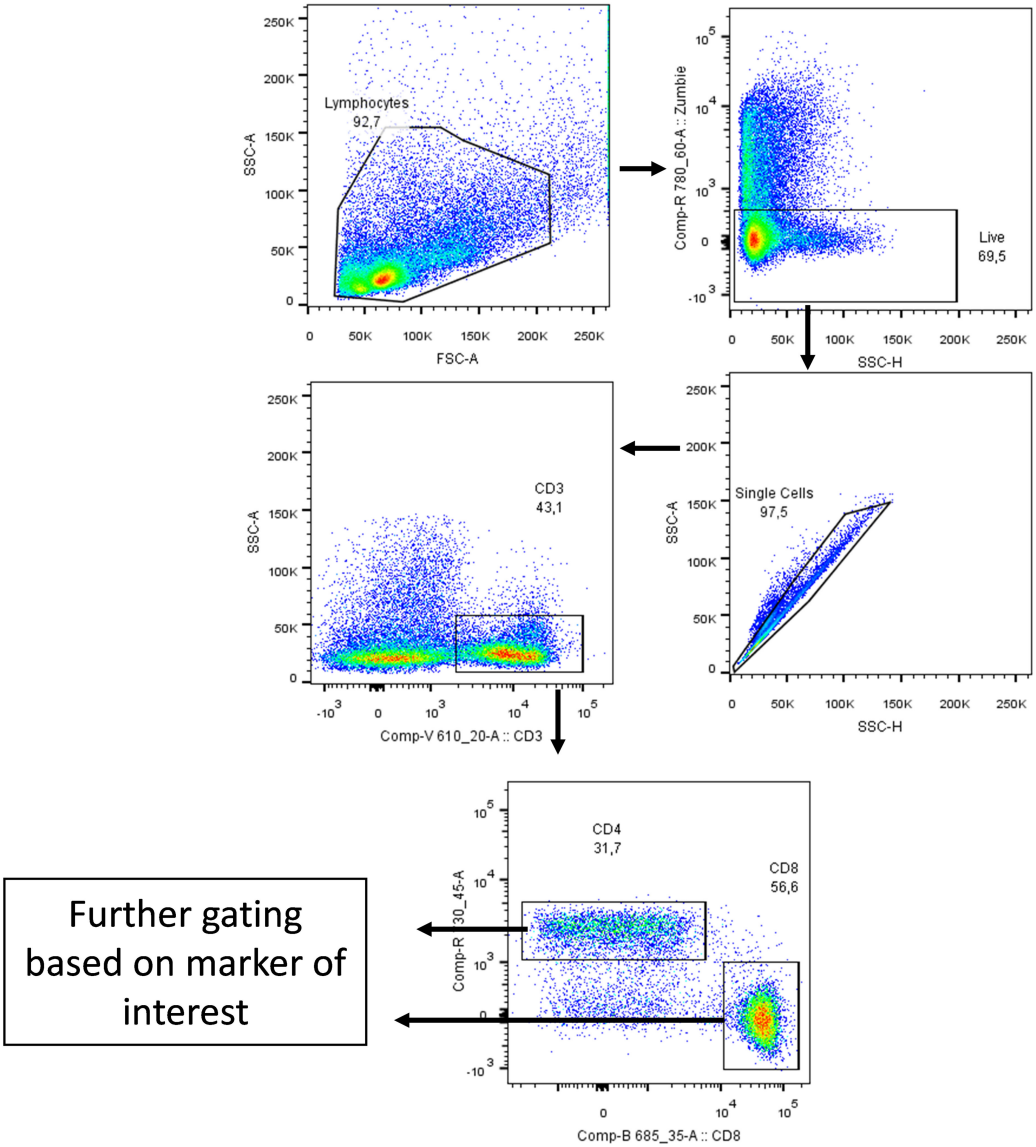
Gating strategy. Cells were gated side scatter area vs forward scatter area then Zombie Aqua dye (which penetrates non-viable cells but not viable cells; Biolegend, San Diego, USA) was used to gate on live cells only. After that, cells were gated on side scatter height vs side scatter area to exclude duplicate cells. Cells were then gated on CD3 marker and then on CD4 and CD8 markers. Lastly, cells were gated based on marker of interest (CD69, FasR, CD137 and CD107a) as shown on the following figures. The arrows indicate stepwise progression through each of the gating steps.

**Figure 3:**
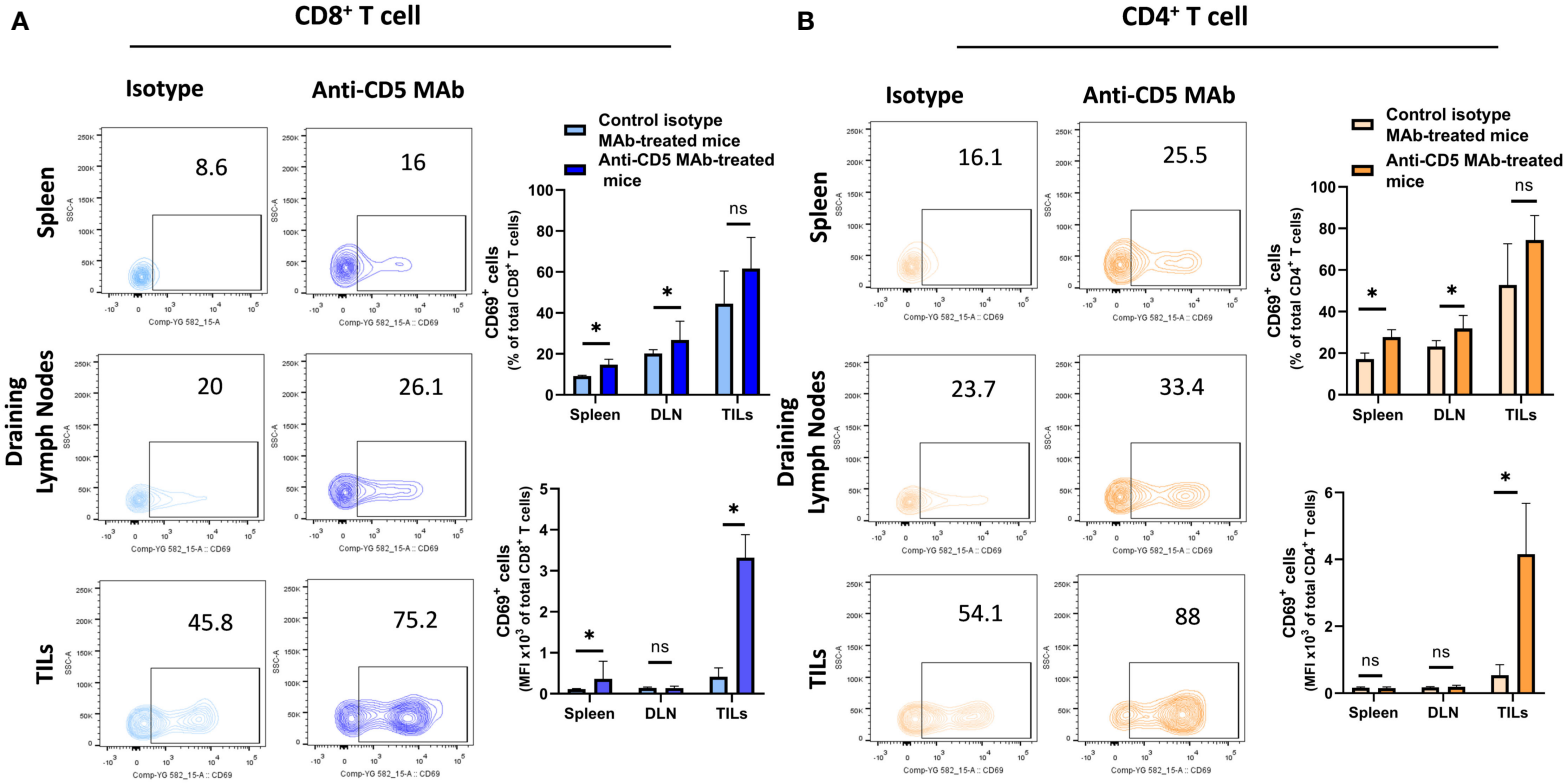
Fraction of CD8^+^/CD69^+^ T and CD4^+^/CD69^+^ T cells after treatment with anti-CD5 Mab *in vivo*. **(A)** The fraction of CD8^+^/CD69^+^ T and MFI of CD69 on CD8^+^ T cell isolated from spleens, draining lymph nodes, and TILs isolated from 4T1 tumour-bearing BALB/c mice treated with anti-CD5 MAb or isotype Mab control. **(B)** The fraction of CD4^+^/CD69^+^ T and MFI of CD69 on CD4^+^ T cell isolated from spleens, draining lymph node, and TILs isolated from 4T1 tumour-bearing BALB/c mice treated with anti-CD5 MAb or control isotype MAb. Data are mean ± SD (*n* = 3 mice), one representative experiment of three. *p < 0.05 (Student’s unpaired one-tailed *t*-test). MFI, mean fluorescence intensity. ns, non significant.

In our previously-reported study we reported that splenocytes stimulated *ex vivo* with anti-CD3/anti-CD28 or 4T1 tumour lysate and treated with anti-CD5 MAb had an increased fraction of CD8^+^CD69^+^ T cells compared to cells stimulated with anti-CD3/anti-CD28 or 4T1 tumour lysate and isotype control *ex vivo* (28). To investigate whether *in vivo* administration of anti-CD5 MAb enhanced T cell activation we isolated spleen, draining lymph nodes, and TILs and assessed the level of CD69 on T cells (**Figure 2** for gating strategy). We observed an increased fraction of CD69^+^CD8^+^ T cells after anti-CD5 Mab treatment in spleen and draining lymph nodes of mice compared to mice treated with isotype control MAb (**Figure 3A**). Furthermore, CD8^+^ TILs isolated from anti-CD5 MAb-treated mice had an increased level of CD69 compared to mice treated with isotype control MAb (**Figure 3A**). Furthermore, we found an increased level of CD69 on CD69^+^CD4^+^ T cells in spleen and draining lymph nodes in anti-CD5 MAb-treated mice (**Figure 3B**). Similar to CD8^+^ TILs, the mean fluorescence intensity (MFI) of CD69 was higher in CD4^+^ TILs in anti-CD5 MAb-treated mice (**Figure 3B**). It is important to note that MFI indicates the degree of CD69 positivity of T cell populations and not the number of CD69^+^ cells and is a measure of activation distinct from assessment of the number of activated cells. These data indicate that treatment with anti-CD5 MAb enhances T cell activation *in vivo*.

### 3.4 Increased level of Fas receptor on T cells after anti-CD5 MAb treatment

**Figure 4:**
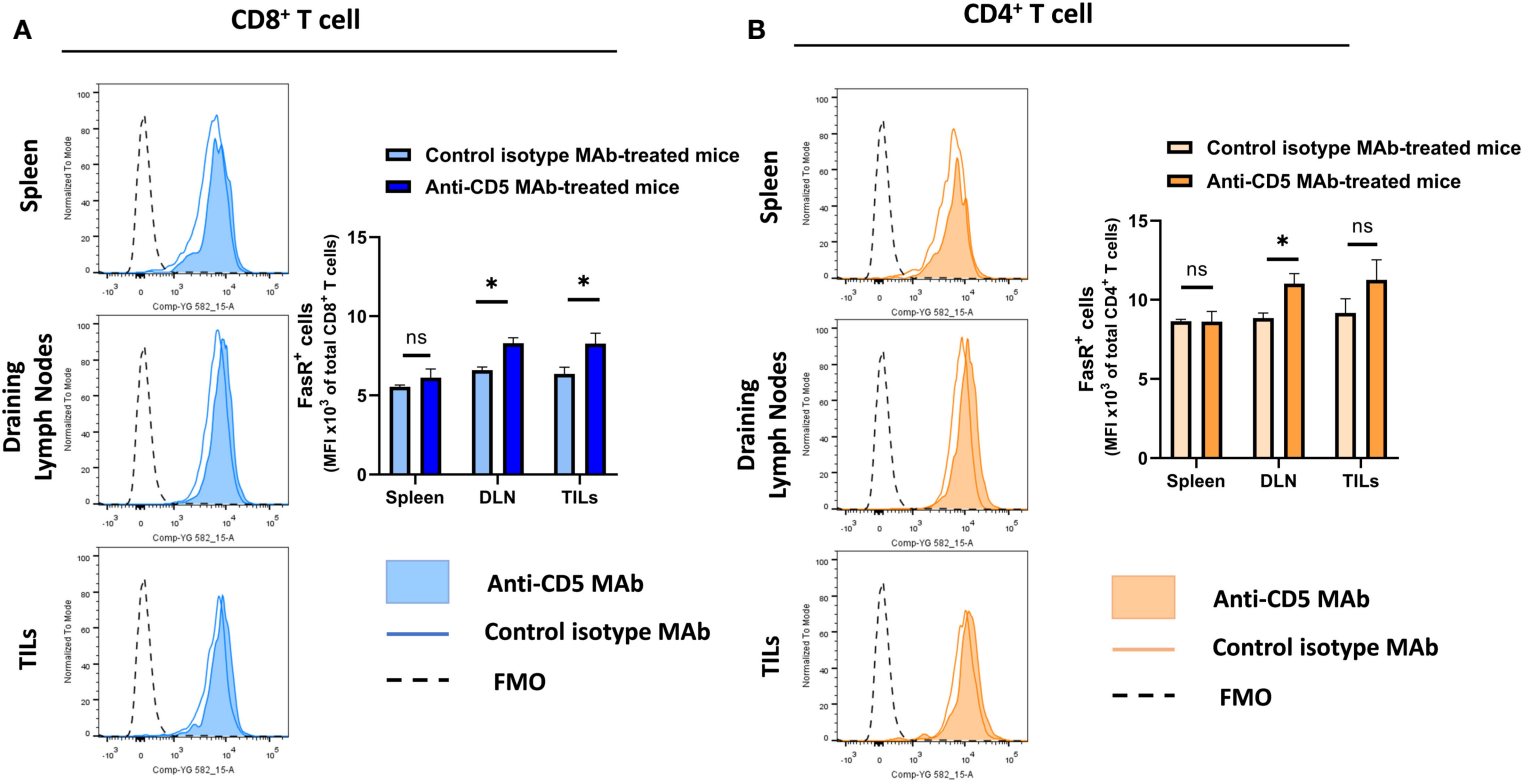
Level of Fas receptor on CD8^+^ T cells and CD4^+^ T cells after treatment with anti-CD5 MAb *in vivo*. **(A)** The MFI of Fas receptor on CD8^+^ T cells isolated from spleens, draining lymph node, and TILs isolated from 4T1 tumour-bearing BALB/c mice that were treated with anti-CD5 MAb or isotype MAb control. **(B)** The MFI of Fas receptor on CD4^+^ T cells isolated from spleens, draining lymph nodes, and TILs isolated from 4T1 tumour-bearing BALB/c mice treated with anti-CD5 MAb or control isotype MAb. Data are mean ± SD (*n* = 3 mice), one representative experiment of three. *p < 0.05 (Student’s unpaired one-tailed *t*-test). MFI, mean fluorescence intensity. FMO, Fluorescence Minus One. ns, non significant.

Increased T cell activation can lead to upregulation of Fas receptor (28, 31). To assess whether anti-CD5 MAb treatment resulted in increased FasR on T cells, cells isolated from spleen, draining lymph node and TILs were stained with anti-Fas receptor MAb to determine the level of Fas receptor on T cells. Anti-CD5 MAb treatment increased Fas receptor levels in CD8^+^ T cells from draining lymph nodes and the tumour microenvironment, but not spleens, of mice (**Figure 4A**). Treatment with anti-CD5 MAb also induced an increased level of Fas on CD4^+^ T cells in draining lymph nodes (**Figure 4B**).

### 3.5 Increased T cell tumour-reactivity and degranulation after treatment with anti-CD5 MAb *in vivo*

**Figure 5:**
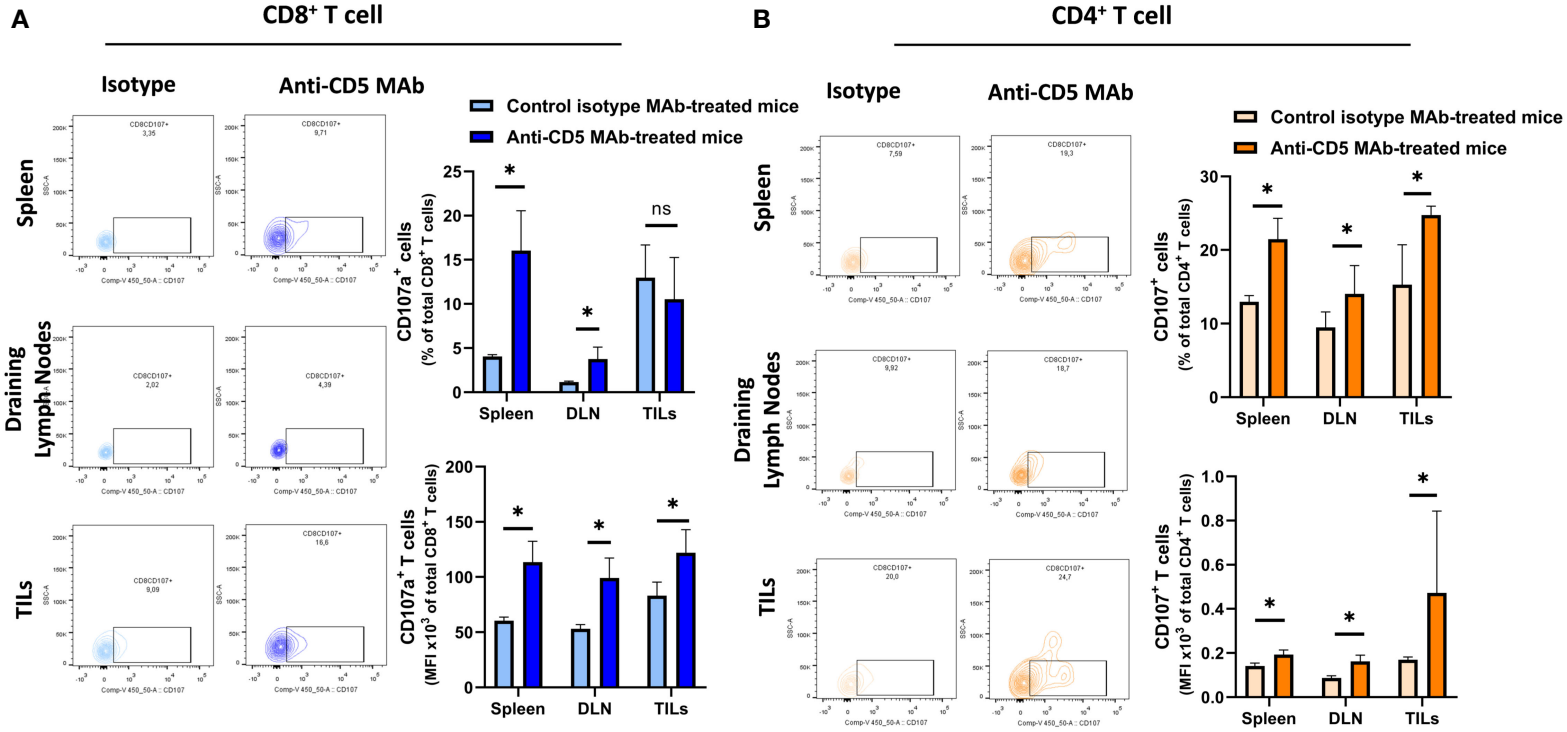
**(A)** The fraction of CD8^+^/CD107a^+^ T cells and MFI of CD107a on CD8^+^ T cells isolated from spleens, draining lymph nodes, and TILs from 4T1 tumour-bearing BALB/c mice treated with anti-CD5 MAb or control isotype MAb. **(B)** The fraction of CD4^+^/CD107a^+^ T and MFI of CD107a on CD4^+^ T cell isolated from spleens, draining lymph nodes, and TILs isolated from 4T1 tumour-bearing BALB/c mice that were treated with anti-CD5 MAb or control isotype MAb. Data are mean ± SD (*n* = 3 mice), one representative experiment of three. *p < 0.05 (Student’s unpaired one-tailed *t*-test). MFI, mean fluorescence intensity. ns, non significant.

**Figure 6:**
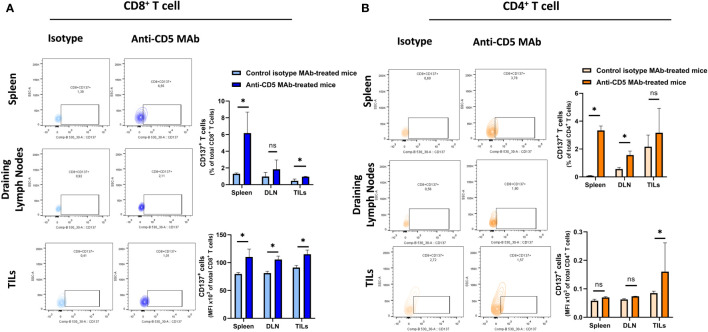
**(A)** The fraction of CD8^+^/CD137^+^ T and MFI of CD137 on CD8^+^ T cells isolated from spleens, draining lymph nodes, and TILs isolated from 4T1 tumour-bearing BALB/c mice that were treated with anti-CD5 MAb or control isotype MAb. **(B)** The fraction of CD4^+^/CD137^+^ T and MFI of CD137 on CD4^+^ T cells isolated from spleens, draining lymph nodes, and TILs isolated from 4T1 tumour-bearing BALB/c mice treated with anti-CD5 MAb or control isotype MAb. Data are mean ± SD (*n* = 3 mice), one representative experiment of three. *p < 0.05 (Student’s unpaired one-tailed *t*-test). MFI, mean fluorescence intensity. ns, non significant.

We further determined the cytotoxic T lymphocyte (CTL) effector function after treatment with anti-CD5 MAb *in vivo*. Here, CTL effector function was assessed by determining the number of CD8^+^ cells positive for CD107a (a surrogate marker for degranulation) (32). An increased fraction of CD107a^+^CD8^+^ T cells among all CD8^+^ T cells were isolated from mice treated with anti-CD5 MAb, compared to mice treated with isotype MAb control in spleens and draining lymph nodes (**Figure 5A** upper panel). The MFI was also higher in spleens, draining lymph nodes, and TILs in anti-CD5 MAb-treated mice (**Figure 5A** lower panel). Furthermore, the fraction of CD107a^+^CD4^+^ T cells and the MFI of CD107a were higher in spleens, draining lymph nodes, and TILs from anti-CD5 MAb-treated mice (**Figure 5B** upper panel and lower panel). In addition, antigen-specific T cells were further assessed by determining the level of CD137, a member of the TNFR-family with costimulatory function and a surrogate marker for antigen-specific activation of T cells (33). The data show an increase in the fraction of CD137^+^CD8^+^ T cells in spleen and TILs (**Figure 6A** upper panel), and a trend (insufficient to indicate significance) toward an increase in the fraction of CD137^+^CD8^+^ T cells in draining lymph nodes (**Figure 6A** upper panel). The MFI of CD137 was higher in CD8^+^ T cells in spleen, draining lymph nodes, and TILs isolated from anti-CD5 MAb-treated mice (**Figure 6A** lower panel). Moreover, anti-CD5 MAb-treated mice had an increased fraction of CD137^+^CD4^+^ T cells in spleen and draining lymph nodes but not in TILs (**Figure 6B** upper panel). The MFI of CD137 was upregulated in CD4^+^ TILs after treatment with anti-CD5 MAb (**Figure 6B** lower panel). Together these data indicate that antigen-specific and effector functions of CD8^+^ T and CD4^+^ T cells are enhanced after treatment with anti-CD5 MAb.

## 4 Discussion

CD5 has been targeted as a tumour antigen expressed by non-solid tumours using depleting, toxin-conjugated anti-CD5 Mab (6–12, 34) and most recently by using CD5 CAR T cells (13–22, 35). It is also upregulated during human T cell activation and negatively regulates the T cell receptor on cytotoxic T cells, limiting their ability to recognize poorly immunogenic tumour antigens (2). We have shown previously that blocking CD5 *ex vivo* resulted in increased CTL activation and tumour cell cytotoxicity (28) and we have shown that T cells with low CD5 levels isolated from tumour homografts in mice exhibit increased activation and effector function (24). Here, we determined whether blocking CD5 *in vivo* could increase activation of non-malignant T cells and enhance detection of poorly immunogenic tumour antigen. 4T1 mouse breast cancer cells were used as a model of poorly immunogenic and highly metastatic triple negative breast cancer in humans (36). These tumours and host mice were used to determine whether anti-CD5 MAb *in vivo* could enhance T cell activation and ability to recognize poorly immunogenic tumour antigen(s). Mice were injected with 4T1 mouse tumour cells and treated with anti-CD5 MAb. The activation and function of T cells isolated from spleen, draining lymph nodes, and tumours were further assessed for markers of activation by flow cytometry.

The data show that administration of anti-CD5 MAb *in vivo* increases the ratio of CD8^+^/CD4^+^ T cells in draining lymph nodes and tumours. CD8^+^ and CD4^+^ T cells were activated in spleens, draining lymph nodes, and TILs, suggesting that anti-CD5 treatment mediates enhanced activation by influencing CD5 effects on TCR signalling. The predominating CD8^+^ T cells and smaller number of CD4^+^ T cells were activated in spleens, draining lymph nodes, and TILs as assessed by the activation marker CD69 (37), suggesting that anti-CD5 treatment mediates enhanced activation by influencing CD5 effects on TCR signalling. Our data are consistent with our previous report showing increased Fas receptor levels in CD8^+^ T cells (a marker of T cell activation) after treatment with anti-CD5 MAb *ex vivo* (28). We observed increased Fas receptor levels on the surface of CD8^+^ T cells isolated from draining lymph nodes and TILs after *in vivo* treatment with anti-CD5 MAb. Because increased Fas receptor levels occur in response to TCR stimulation (31), treatment with anti-CD5 MAb may led to TCR sensitivity to tumour antigen and resulting increased activation. These results suggest that treatment with anti-CD5 MAb could also enhance the effector function of T cells. We found higher effector function in both draining lymph nodes and TILs, as shown by increased levels of surrogate markers of T cell degranulation and antigen-specific T cell activation (CD107a and CD137, respectively). Together, these data suggest that treatment with non-depleting anti-CD5 MAb *in vivo* increases T cell activation and effector function.

The implications of increased markers of activation in multiple populations of T cells (in spleen and far from the site of anti-CD5 injection and implanted 4T1 tumours; in draining lymph nodes likely to contain white blood cells that have made contact with tumours; and in the tumour microenvironment itself [TILs]) suggests that anti-CD5 therapy has the potential to promote anti-tumour T cell activity, not only at the site of individual tumours in close proximity to the site of injection with anti-CD5 molecules, but systemically. Systemic rather than localized anti-tumour T cell activity would be expected to have greater capacity to inhibit and/or ablate growth of tumours at non-primary sites, possibly including metastatic tumours.

Administration of anti-CD5 *in vivo* results in increased numbers of CD8^+^ cells relative to CD4^+^ T cells and enhanced CD8^+^ T cell activation and effector function. To assess whether administration of anti-CD5 MAb can delay tumour growth, anti-CD5 MAb was injected peritumorally (as a strategy to maximize antitumor activity while potentially limiting systemic overactivation of T cell and the risk excessive systemic activation-induced cell death). The result shows delayed tumour growth after treatment with anti-CD5 MAb.

Although in our model we did not observe severe immune-related adverse events, the use of therapeutic antibodies can induce such a reaction in humans (38–40). It is important to manage such events to maintain treatment efficacy (41–43). Furthermore, administration of antibodies as drugs may be challenging due to their potential to induce production of anti-antibodies. It may be useful to employ delivery vehicles such as exosomes (44–46) or nanoparticles (47–49) in the future to diminish that potential.

The use of toxin-conjugated anti-CD5 depleting MAb to treat CD5^+^ non-solid tumours has been reported (14, 50). However, the impact of CD5 blocking antibody on normal T cells in CD5^-^ solid tumour has not been well-studied. Despite one study showing administration of CD5 polyclonal antibodies to slow the growth of EL lung cancer (51) there has been no further report of administration of anti-CD5 MAb in normal T cells *in vivo*.

Overall, this study is the first to illustrate changes in immune cell subsets in lymphoid organs as well as in the tumour microenvironment after *in vivo* administration of anti-CD5 MAb. In addition, it illustrates the phenotypic changes that resulted from anti-CD5 MAb *in vivo* and its capacity to delay tumour growth. These results warrant further investigation of anti-CD5 MAb as an anticancer immunotherapy, including in combination with other current anti-tumour immunotherapies.

## Data availability statement

The original contributions presented in the study are included in the article/**Supplementary Material**. Further inquiries can be directed to the corresponding author.

## Ethics statement

The animal study was reviewed and approved by the Animal Use Subcommittee of the University of Western Ontario. The studies were conducted in accordance with the local legislation and institutional requirements.

## Author contributions

FA: Conceptualization, Formal analysis, Funding acquisition, Investigation, Methodology, Writing – original draft, Writing – review & editing. W-PM: Conceptualization, Formal analysis, Investigation, Project administration, Supervision, Writing – review & editing. JK: Funding acquisition, Investigation, Methodology, Project administration, Resources, Supervision, Validation, Visualization, Writing – review & editing.

## Supplementary material

Supplementary Figure 1 Body weight of mice after treatment with isotype control MAb and anti-CD5 MAb (200 μg/mouse). Mice were injected with antibodies 7 days post tumour injection and every three to four days thereafter for a total of four injections. Body weights were measured every three to four days until euthanizing the mice.
