# Supplementary material for: CD5 blockade, a novel immune checkpoint inhibitor, enhances T cell anti-tumour immunity and delays tumour growth in mice harbouring poorly immunogenic 4T1 breast tumour homografts
Source: Front Immunol. 2024 Feb 29;15:1256766. doi: 10.3389/fimmu.2024.1256766 (PMC10937348; doi:10.3389/fimmu.2024.1256766)
Supplement: Supplementary Figure 1 — Body weight of mice after treatment with isotype control MAb and anti-CD5 MAb (200 μg/mouse). Mice were injected with antibodies 7 days post tumour injection and every three to four days thereafter for a total of four injections. Body weights were measured every three to four days until euthanizing the mice. [file Presentation_1.pptx]

## Slide 1
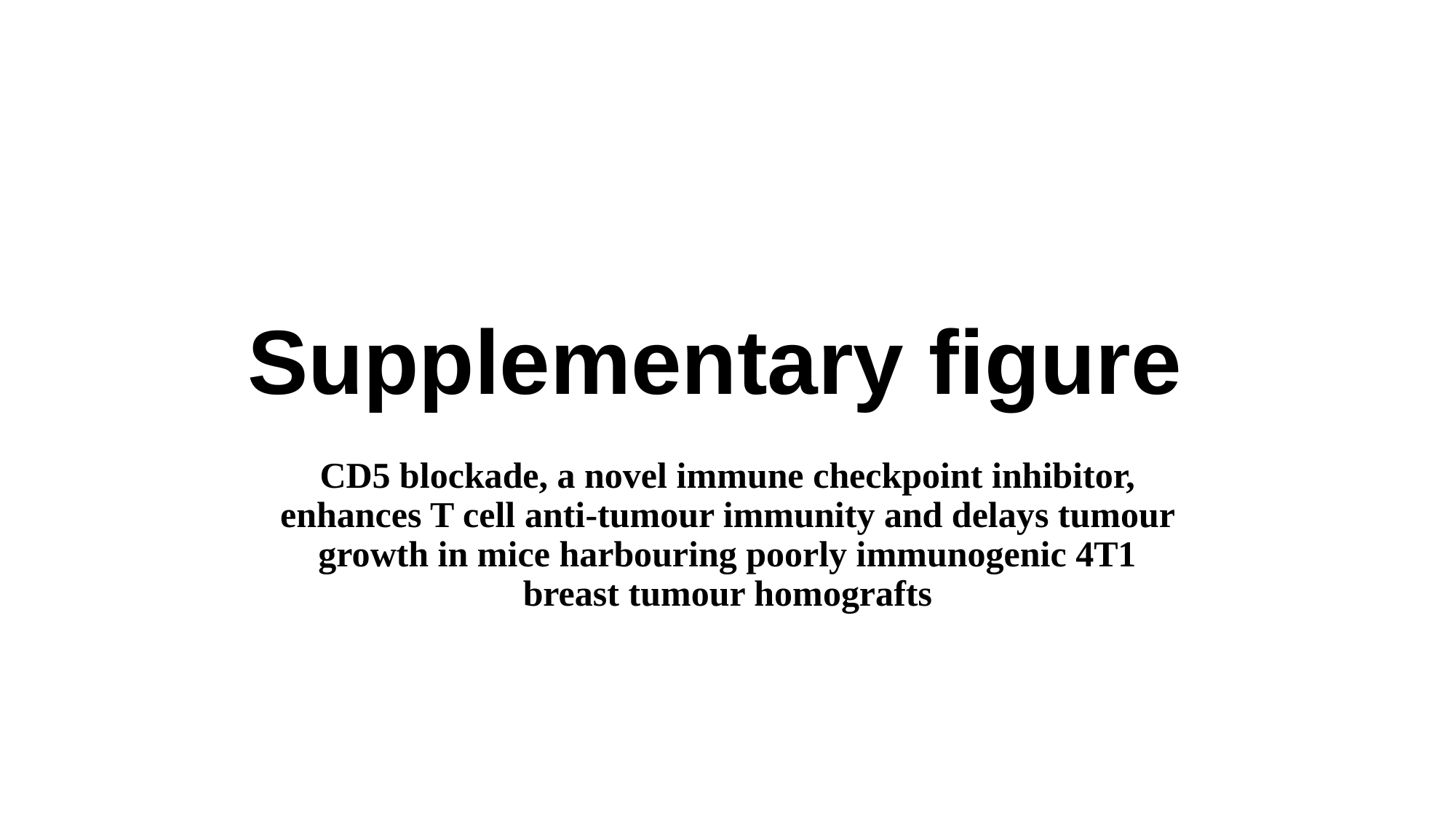

# Supplementary figure
CD5 blockade, a novel immune checkpoint inhibitor, enhances T cell anti-tumour immunity and delays tumour growth in mice harbouring poorly immunogenic 4T1 breast tumour homografts

## Slide 2
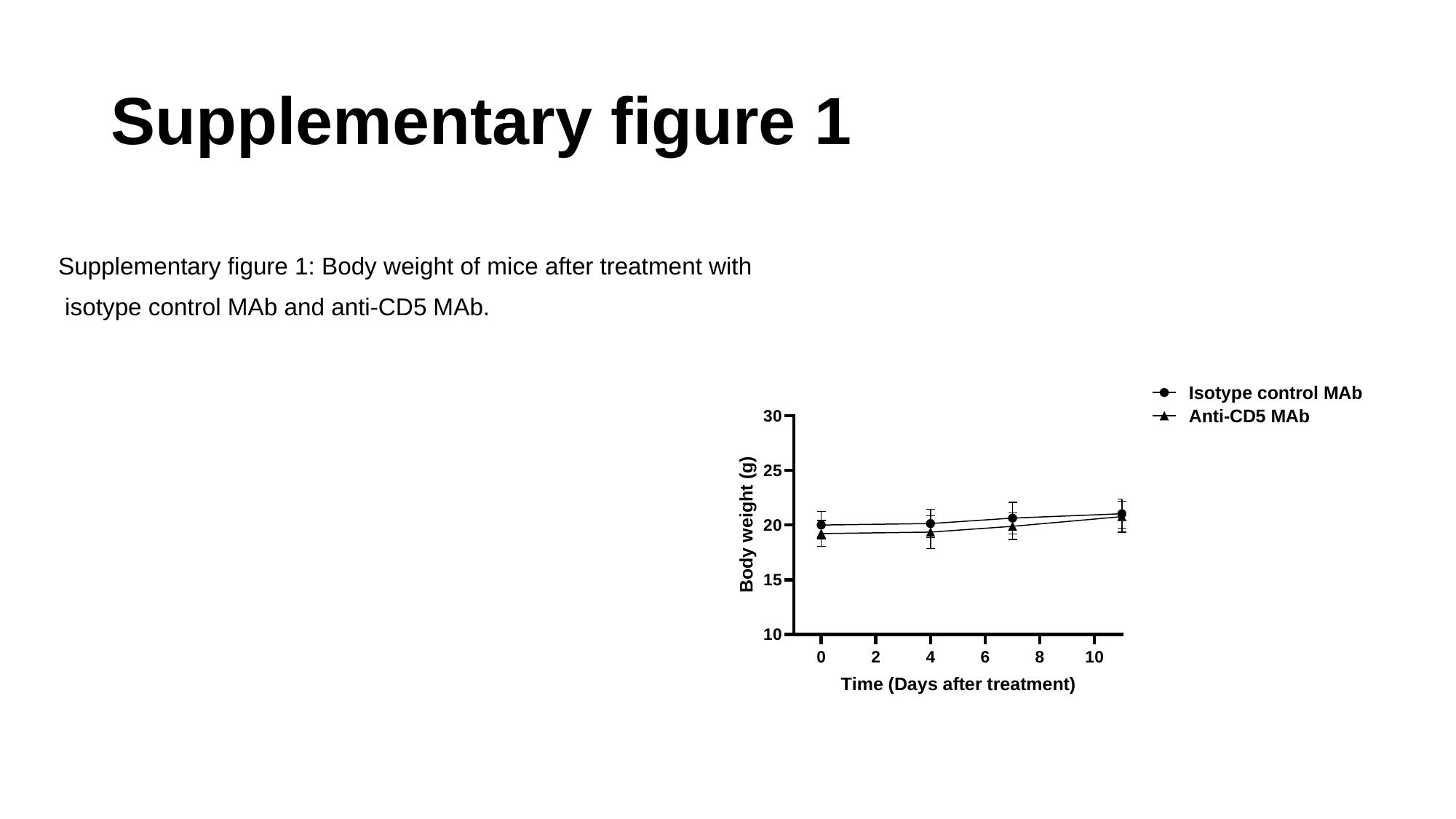

# Supplementary figure 1
Supplementary figure 1: Body weight of mice after treatment with
 isotype control MAb and anti-CD5 MAb.
